# Residues T_48_ and A_49_ in HIV-1 NL4-3 Nef are responsible for the counteraction of autophagy initiation, which prevents the ubiquitin-dependent degradation of Gag through autophagosomes

**DOI:** 10.1186/s12977-021-00576-y

**Published:** 2021-10-28

**Authors:** Sergio Castro-Gonzalez, Yuexuan Chen, Jared Benjamin, Yuhang Shi, Ruth Serra-Moreno

**Affiliations:** 1 Department of Chemistry, Umeå University, Umeå, Sweden; 2 Microbiology and Immunology, University of Rochester Medical Center, Rochester, NY USA

**Keywords:** Autophagy, HIV-1, Nef, Gag

## Abstract

**Background:**

Autophagy plays an important role as a cellular defense mechanism against intracellular pathogens, like viruses. Specifically, autophagy orchestrates the recruitment of specialized cargo, including viral components needed for replication, for lysosomal degradation. In addition to this primary role, the cleavage of viral structures facilitates their association with pattern recognition receptors and MHC-I/II complexes, which assists in the modulation of innate and adaptive immune responses against these pathogens. Importantly, whereas autophagy restricts the replicative capacity of human immunodeficiency virus type 1 (HIV-1), this virus has evolved the gene *nef* to circumvent this process through the inhibition of early and late stages of the autophagy cascade. Despite recent advances, many details of the mutual antagonism between HIV-1 and autophagy still remain unknown. Here, we uncover the genetic determinants that drive the autophagy-mediated restriction of HIV-1 as well as the counteraction imposed by Nef. Additionally, we also examine the implications of autophagy antagonism in HIV-1 infectivity.

**Results:**

We found that sustained activation of autophagy potently inhibits HIV-1 replication through the degradation of HIV-1 Gag, and that this effect is more prominent for *nef*-deficient viruses. Gag re-localizes to autophagosomes where it interacts with the autophagosome markers LC3 and SQSTM1. Importantly, autophagy-mediated recognition and recruitment of Gag requires the myristoylation and ubiquitination of this virus protein, two post-translational modifications that are essential for Gag’s central role in virion assembly and budding. We also identified residues T_48_ and A_49_ in HIV-1 NL4-3 Nef as responsible for impairing the early stages of autophagy. Finally, a survey of pandemic HIV-1 transmitted/founder viruses revealed that these isolates are highly resistant to autophagy restriction.

**Conclusions:**

This study provides evidence that autophagy antagonism is important for virus replication and suggests that the ability of Nef to counteract autophagy may have played an important role in mucosal transmission. Hence, disabling Nef in combination with the pharmacological manipulation of autophagy represents a promising strategy to prevent HIV spread.

**Supplementary Information:**

The online version contains supplementary material available at 10.1186/s12977-021-00576-y.

## Background

Macroautophagy (hereafter autophagy) is a degradative process essential for cellular homeostasis characterized by the recruitment and delivery of intracellular targets to lysosomes for their degradation. Autophagy is activated under situations of stress such as starvation or infections [1–4] and results in the engulfment of autophagic cargo by specialized double-membrane vesicles (autophagosomes), which eventually fuse with lysosomes to facilitate the degradation of their content. Remarkably, the autophagy machinery is highly conserved among all eukaryotic organisms, which already denotes the relevance of this pathway [5, 6]. Involving more than 30 autophagy-related genes, the autophagic process takes place in three consecutive steps: initiation, elongation and maturation. First, upon activation by a plethora of stress stimuli, the nucleation of a double-membrane structure called phagophore takes place. The phagophore is used as a primer for the biogenesis of autophagosomes [2, 7]. This step requires the formation of the multimolecular enzymatic complex class III phosphatidylinositol 3 kinase complex 1 (Class III PtdIns3K C1) that contains the protein Beclin1/BECN1, an essential initiator of autophagy [8, 9]. Second, the class III PtdIns3K C1 not only facilitates phagophore nucleation, but also elongation of this membranous structure through the recruitment of an E3-like enzymatic complex that mediates a key reaction in the autophagy pathway: the conjugation of phosphatidylinositol ethanolamine (PE) to the microtubule associated protein 1 light chain 3 (MAPILC3 or LC3). The lipidation of LC3 converts its inactive and cytosolic isoform, LC3-I, into the autophagy-competent LC3-II variant, which is able to associate with both, the inner as well as the outer membrane of autophagosomes. Membrane-associated LC3-II plays a critical role in the elongation of autophagosomes and the recruitment of cargo for degradation [6, 10, 11]. Targets for autophagy elimination are usually poly-ubiquitinated proteins that are recognized and recruited by different autophagy receptors or adaptor proteins, which serve as a bridge between ubiquitin-tagged substrates and LC3-II molecules found on the inner membrane of elongating autophagosomes [12–14]. In this regard, sequestosome-1 (SQSTM1, also known as p62) is one of the main adaptor proteins, which, besides being responsible for substrate recognition and sequestration, also provides structural support for the formation and final enclosure of autophagosomes [12, 15, 16]. Finally, the last stage of autophagy involves the fusion between the autophagosome and a lysosome creating the so-called autophagolysosome or autolysosome. This fusion event leads to the degradation of the autophagic cargo due to the action of the lysosomal acid hydrolases [17, 18].

In addition to its role in cellular quality control and its contribution as an alternative source of energy under starvation conditions, autophagy has recently risen as a noteworthy asset in the innate defense against intracellular pathogens, such as viruses [19–21]. From this perspective, not only does autophagy promote the direct elimination of cytosolic viral components, but also boosts immune responses by using the residual products generated by autophagolysosomal degradation to engage endosomal pattern recognition receptors. In addition, these degradation products can be loaded onto MHC-I and MHC-II molecules to promote antigen-presentation [19, 22, 23]. Hence, autophagy activation plays a crucial role in eliciting both innate and adaptive responses against viruses. In line with these antiviral actions, we recently uncovered that autophagy restricts Human Immunodeficiency Virus type 1 (HIV-1). However, this virus has evolved Nef, a well-known immune evasion factor [24–27], as a countermeasure for autophagy restriction. Particularly, autophagy significantly restricts virion production, and this is associated with reduced levels of the HIV structural protein Gag (p55) [28]. Considering that Gag is the precursor of the capsid protein (p24), and more importantly, is the main driver of virion assembly and budding, we reasoned that autophagy-mediated Gag clearance results in a defect in virion production. As for the ability of Nef to counteract this restriction, our study revealed that, besides its previously reported ability to prevent autophagosome-lysosome fusion [29–31], Nef enhances the association between the initiator of autophagy BECN1 and its natural inhibitor BCL2, preventing LC3 lipidation and halting autophagosome biogenesis. These findings indicate that besides its already known role at blocking late events in the autophagic cascade, Nef additionally intersects with autophagy initiation. Furthermore, this antagonistic effect of Nef is conserved across pandemic clades of HIV-1. Therefore, counteraction of autophagy may have been advantageous for viral infectivity, and thus, the successful spread of HIV-1 worldwide [28].

In this study, we sought to characterize the genetic determinants that govern the mutual antagonism between HIV and autophagy. First, we confirmed that HIV Gag is directed to autophagy-mediated elimination through a mechanism that involves Gag ubiquitination and its capacity to associate with membranes. Second, our mapping analyses uncovered that residues T_48_ and A_49_ within the N-terminal domain of NL4-3 Nef are essential for Nef’s ability to suppress the early stages of autophagy. Remarkably, mutation of these residues has no significant effects on Nef’s ability to block autophagy maturation or other activities of Nef such as MHC-I, CD4 or SERINC5 down-regulation, indicating that autophagy antagonism is genetically separable from other roles of Nef. Additionally, a survey of transmitted/founder (T/F) pandemic HIV-1 viruses revealed that autophagy antagonism is a common trait among these isolates. T/F viruses are highly infectious HIV variants that are commonly studied to identify phenotypic properties associated with increased viral transmission and infectivity [32–36]. Hence, the high resistance of these clones to autophagy restriction suggests that autophagy antagonism is important for infectivity and spread.

## Results

### The pharmacological activation of autophagy restricts HIV replication

**Fig. 1:**
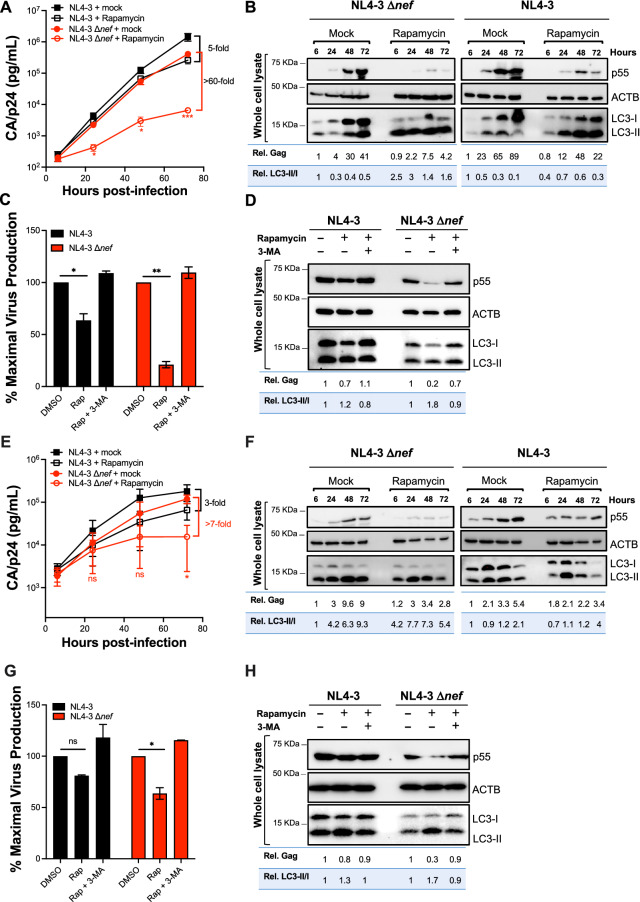
Autophagy restricts HIV replication in Jurkat and primary CD4^+^ T cells. **A**, **B** Jurkat cells and **E**, **F** primary CD4^+^ T cells were infected with HIV-1 NL4-3 or NL4-3 Δ*nef* and treated with rapamycin (6.5 μM) for 3 days. Supernatants were collected at each time point and were analyzed by p24 antigen-capture ELISA to determine relative viral replication. Jurkat cells (**C**, **D**) and primary CD4^+^ T cells (**G**, **H**) were infected with HIV-1 NL4-3 or NL4-3 Δ*nef* and treated with DMSO, rapamycin (6.5 μM) or rapamycin and 3-MA (3 mM) for 6 h. Next, supernatants were collected and analyzed by p24 antigen-capture ELISA to determine relative viral replication. Data represents the mean and SEM of three independent replicates and significantly different values are indicated by asterisks (*P ≤ 0.05; **P ≤ 0.01; ***P ≤ 0.001). Cell lysates from Jurkat cells (**B**, **D**) and primary CD4^+^ T cells (**F**, **H**) were analyzed by western blotting for Gag/p55, LC3-I/LC3-II and ACTB (β-actin) for each time point. Blots are representative of three independent experiments

In our previous study, we reported that short-term treatment with the autophagy-activating drug rapamycin negatively impacts virion release for *nef*-deficient HIV-1 NL4-3 in many cell types, including HEK293T, THP-1-derived macrophages, primary CD4^+^ T cells and Jurkat CD4^+^ T cells [28]. To evaluate the effect that autophagy poses on HIV fitness over several rounds of replication, we infected Jurkat cells with either HIV-1 NL4-3 or NL4-3 Δ*nef* and maintained a constant concentration of rapamycin (6.5 μM) for 72 h. Virus replication was monitored every 24 h by measuring the levels of the capsid protein p24 (CA) released to the supernatant by p24 antigen-capture ELISA. Whereas rapamycin caused a 5-fold reduction in the replication of wild type NL4-3 72 h post-infection, this effect was magnified for the *nef*-defective virus, resulting in a 60-fold defect in its replication kinetics (Fig. 1A). Western blot analyses of the cell lysates showed that rapamycin treatment promoted the conversion of LC3-I into LC3-II (which serves as a measure for autophagy flux), confirming that autophagy was effectively activated. Besides this effect on LC3, rapamycin caused a concomitant defect in the emergence of Gag (p55) (Fig. 1B). However, in line with our previous findings [28], the presence of Nef counteracted both reduction of Gag levels and autophagy flux. This last effect is evidenced by the fact that, even in the presence of rapamycin, cells infected with wild type NL4-3 exhibited an accumulation of LC3-I, whereas in the Δ*nef*-infected cells autophagy flux proceeded normally (Fig. 1B; see quantifications). To verify that rapamycin-induced autophagy is responsible for the defect in Gag levels and, in consequence, in virion production, similar assays were performed in the presence of 3-methyladenine (3-MA), a drug that blocks autophagy initiation [37, 38]. Since this compound can also trigger autophagy when used during prolonged treatments [39], 3-MA and rapamycin were added to the cultures for only 6 h. In this case, virion production was expressed as the percentage of maximal release relative to the DMSO-treated samples. Consistent with our previous work [28], addition of 3-MA prevented the activation of autophagy mediated by rapamycin, consequently rescuing virion production (Fig. 1C, D). To further evaluate the physiological relevance of these observations, we performed parallel assays in primary CD4^+^ T cells obtained from three healthy donors. Similar to the results obtained in Jurkat cells, rapamycin treatment successfully restricted HIV replication. Once again, the impact on virus replication was associated with an increase in autophagy flux and a defect in the emergence of Gag over time. Although the degree of restriction was not as striking as in the Jurkat system, autophagy activation posed a bigger hurdle for HIV Δ*nef* than for wild type HIV (Fig. 1E, F). Likewise, treatment with 3-MA prevented autophagy activation and rescued Gag and virion levels (Fig. 1G, H). Hence, these findings indicate that the pharmacological activation of autophagy limits HIV replication in T cell lines and primary CD4^+^ T cells, and further confirm that Nef is an autophagy antagonist.

### Autophagy specifically targets HIV Gag for autolysosomal degradation

**Fig. 2:**
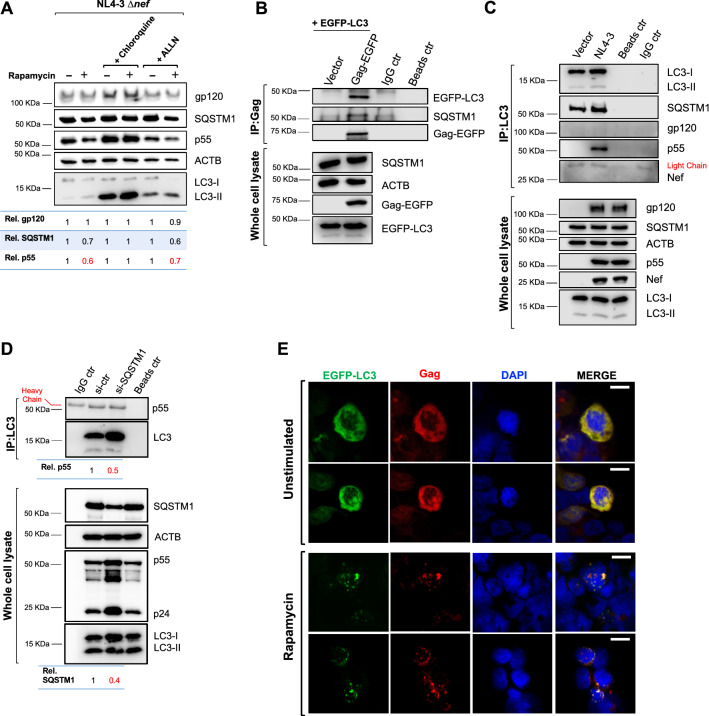
Autophagy targets HIV Gag for degradation. **A** HEK293T cells were transfected with HIV-1 NL4-3 Δ*nef* proviral DNA and treated with rapamycin (4 μM), chloroquine (60 μM) and/or ALLN (25 μM) for 12 h. 48 h later, lysates were analyzed by western blot for gp120, SQSTM1, p55, ACTB, and LC3. Densitometric analyses were performed to determine the relative ratios of gp120, SQSTM1 and p55. **B** HEK293T cells were co-transfected with *Gag*-EGFP, EGFP*-LC3B* or an empty vector. 48 h later, cells were harvested, and Gag was immunoprecipitated. The pulldown fraction was examined for SQSTM1 and LC3. Lysates were also analyzed by western blot for SQSTM1, Gag, LC3 and ACTB. **C** HEK293T cells were transfected with the HIV-1 NL4-3 provirus or an empty retroviral vector. 48 h later, cells were harvested, and LC3 was immunoprecipitated. The pulldown fraction was examined for LC3, SQSTM1, p55, gp120 and Nef. Lysates were also analyzed by western blot for gp120, SQSTM1, p55, Nef, LC3 and ACTB. **D** HEK293T cells treated with an irrelevant siRNA (si-ctr) or SQSTM1-specific siRNAs were transfected with HIV-1 NL4-3 Δ*nef* proviral DNA. 48 h post-transfection, cells were harvested and endogenous LC3 was immunoprecipitated. The pulldown fraction was examined for LC3 and p55. Lysates were analyzed by western blot for SQSTM1, p55, LC3 and ACTB. **E** HEK293T cells were co-transfected with EGFP*-LC3B* and HIV-1 NL4-3 Δ*nef* proviral DNA. Cells were exposed for 4 h to rapamycin (4 μM) or DMSO prior to microscopy visualization for EGFP-LC3 (green), Gag (red) and the nuclei (blue). Scale bar: 10 μm. All images are representative of three independent experiments

Our results indicate that autophagy represents an important barrier for HIV replication. Therefore, we sought to determine what specific event in the virus life cycle autophagy is intersecting. The data presented in Fig. 1 indicate that autophagy activation is associated with a reduction in Gag (p55). Since Gag plays a crucial role in the recruitment of components for virion assembly [40–42], we hypothesized that autophagy causes defects in particle biogenesis by targeting Gag for elimination. Because autophagy maturation involves the fusion between autophagosomes and lysosomes, where the acidic pH and the presence of specialized proteases cause cargo degradation, we first assessed whether the pharmacological inhibition of lysosomal function could prevent the rapamycin-associated depletion of Gag. For this, we used HEK293T cells, since our previous work demonstrated that the restrictive role of autophagy on Gag levels and virion production—as well as the counteracting effect of Nef—is observed regardless of the cell type investigated, even in primary cells [28]. In addition, HEK293T cells are easier to manipulate, which is an advantage for mechanistic studies. Cells were transfected with the HIV-1 NL4-3 ∆*nef* proviral DNA, since this clone is more susceptible to autophagy restriction. 24 h later, the medium was replaced, and cells were treated with rapamycin for 12 h in the presence and absence of chloroquine (lysosomal inhibitor [43]). To rule out any potential degradation of Gag through the proteasomal pathway, we also included cells treated with rapamycin and ALLN, a proteasomal inhibitor [44]. As previously observed, Gag levels were reduced in cells treated with rapamycin (Fig. 2A; lane 1 vs. 2). The addition of chloroquine effectively blocked autophagy maturation, reflected by a significant accumulation of LC3-II. LC3-II coats the internal and external membrane of autophagosomes. Hence, upon fusion with lysosomes, LC3-II molecules on the internal membrane, as well as LC3-associated autophagy receptors (i.e., SQSTM1), are susceptible to degradation. However, impairment of lysosomal function prevents this process, consequently increasing the overall levels of LC3-II and SQSTM1. Under these experimental conditions, not only was LC3-II and SQSTM1 degradation prevented but also the rapamycin-induced degradation of Gag (Fig. 2A; lanes 3 and 4). By contrast, treatment with ALLN, had no impact on the rapamycin-dependent degradation of Gag or SQSTM1 (Fig. 2A; lanes 5 and 6). Therefore, these results confirm that the reduction of Gag caused by rapamycin is due to increased autophagolysosomal activity. Nevertheless, since HIV Gag is associated to cellular membranes through its myristoylated group in the N-terminus, it is plausible that the presence of Gag in autophagosomes may be coincidental, as a consequence of its membrane distribution. To rule this out, we assessed whether rapamycin-induced autophagy had a similar effect on the levels of gp120, another HIV protein that associates to cellular membranes. Remarkably, autophagy did not promote the degradation of gp120 (Fig. 2A), suggesting that autophagy targets Gag for degradation in a specific manner.

Next, the role of autophagy in targeting Gag for elimination was investigated by co-immunoprecipitation (co-IP) as well as fluorescence microscopy studies. We reasoned that if Gag is redistributed to autophagosomes by means of an autophagy receptor, we should detect a physical interaction—even if it is indirect—between LC3 and HIV Gag. For this, HEK293T cells were co-transfected with the fusion proteins Gag-EGFP and EGFP-LC3. 48 h later, cells were harvested, and lysates were subjected to immunoprecipitation. In this case Gag-EGFP was pulled down with a Gag-specific antibody, and its association with EGFP-LC3 and SQSTM1 was then analyzed (Fig. 2B). Of note, although the Gag and LC3 constructs both contain EGFP, IPs and blots were performed with antibodies against Gag and LC3 and not against EGFP. In order to evaluate if the proteins present in the pulldown fraction were the result of unspecific binding with the magnetic beads employed in these IPs, a control consisting of the cell lysates plus the magnetic beads, but no antibody, was included (beads ctr). Also, to discriminate between the heavy and light chains of the antibody used in the IP and the proteins of interest, an IgG control consisting of lysis buffer and beads coated with the antibody (IgG ctr) was included. Our data revealed that Gag-EGFP interacts with the autophagosome-associated proteins EGFP-LC3 and SQSTM1 (Fig. 2B). This indicates that Gag is recruited to LC3-coated autophagosomes, possibly by means of the adaptor protein SQSTM1. However, because in these assays we used Gag and LC3 constructs fused with EGFP, and EGFP is well known to dimerize [45, 46], we performed a similar experiment, with a more physiological approach, to verify that association between Gag and LC3 is not an artifact due to EGFP oligomerization. For this, endogenous LC3 was immunoprecipitated from HEK293T cells transfected with the full length wild-type NL4-3 provirus or an empty retroviral vector control. Unlike for Fig. 2A, we used the *nef*-competent NL4-3 clone to further examine whether viral proteins that inherently associate with cellular membranes, particularly by means of myristoylation (i.e., Gag, Nef), localize in autophagosomes coincidentally. In this case, the pulldown fraction was analyzed for the presence of SQSTM1, Gag, gp120 and Nef. As anticipated, the pool of LC3-interacting partners was positive for both SQSTM1 and Gag (Fig. 2C). However, no interactions between LC3 and Nef or gp120 were detected, which supports the notion that the autophagy-mediated recruitment of Gag is specific (Fig. 2C). Finally, to determine whether SQSTM1 plays a role in the elimination of Gag, similar assays were performed in cells depleted of this autophagy receptor. For this, HEK293T cells were transfected with a control siRNA or siRNAs specific for SQSTM1 1 day prior to the transfection with the NL4-3 ∆*nef* provirus. 72 h later, cells were harvested, lysates were immunoprecipitated for LC3 and its association with Gag analyzed. Of note, the heavy chain of the antibody used in the IPs was detected in the IgG control sample, but it exhibits a different migration pattern than that of Gag (Fig. 2D, left lane). Whereas similar amounts of Gag were found in the pulldown fraction of both control and SQSTM1-knocked down cells, the amount of LC3-I present in the immunoprecipates of cells depleted of SQSTM1 was remarkably high. Hence, binding between Gag and LC3-I/II relative to the control was reduced by 50% in this cellular context. In addition, due to the lower SQSTM1 levels, and thus slower autophagy flux, the overall amount of Gag/p55 and CA/p24 in the whole cell lysates was higher than in the control cells (Fig. 2D). Yet, the amount of Gag that was immunoprecipitated was not proportionally enriched, which further supports the notion that the recruitment and elimination of Gag through autophagy is greatly influenced by SQSTM1. Remarkably, the fact that LC3-I is the most abundant variant pulled down in the IPs (Fig. 2C, D) indicates that Gag might interact with nonlipidated LC3.

The recruitment of Gag to autophagosomes was demonstrated by fluorescence microscopy analyses. For this, HEK293T stably expressing EGFP-LC3 were transfected with NL4-3 ∆*nef* proviral DNA. Gag was visualized using an Alexa-568 conjugated (red) secondary antibody and the nuclei was stained with DAPI (blue). In the absence of rapamycin, EGFP-LC3 displays a cytosolic distribution. However, upon autophagy activation, it becomes incorporated into nascent autophagosomes and is subsequently detected as green puncta [10]. Consistent with the notion that Gag might interact with LC3-I, Gag was found scattered throughout the cytoplasm following a similar pattern as the cytosolic EGFP-LC3. However, after rapamycin treatment Gag exhibited a punctuate localization highly overlapping with LC3-coated autophagosomes (Fig. 2E). Similar observations were obtained in Jurkat cells (not shown). Hence, these findings confirm that upon autophagy activation HIV Gag is targeted to autophagosomes.

### Ubiquitination and myristoylation are required to target Gag for autophagy-mediated degradation

**Fig. 3:**
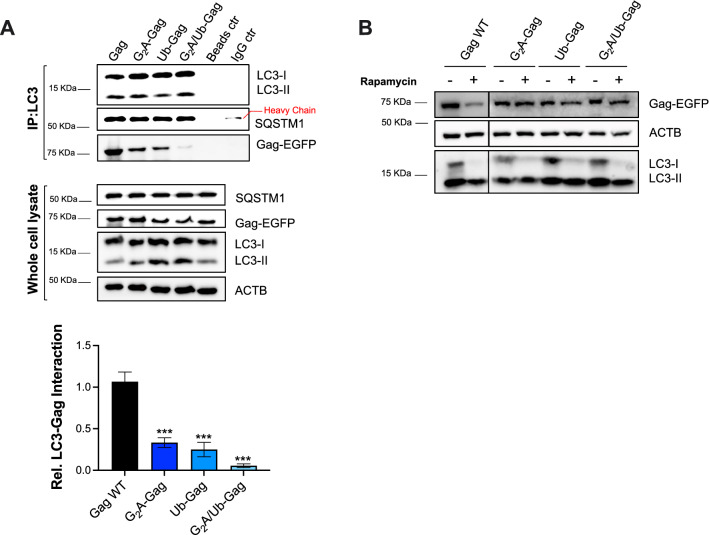
Gag ubiquitination and membrane association are required to target Gag for autophagy elimination. **A** HEK293T cells were transfected with *Gag*, G_2_A-*Gag*, Ub-*Gag* or G_2_A/Ub-*Gag*. 48 h later, cells were harvested and LC3 immunoprecipitated. The pulldown fraction was examined for LC3, SQSTM1 and Gag. Lysates were also analyzed by western blot for the levels of SQSTM1, Gag, LC3 and ACTB. Bottom graph: Densitometric analyses to determine the relative LC3-Gag interaction. Data represent the mean and SEM of 3 independent replicates. **B** HEK293T cells were transfected with *Gag*, G_2_A-*Gag*, Ub-*Gag* or G_2_A/Ub-*Gag* and treated with rapamycin (4 μM) for 12 h. The cell lysates were analyzed by western blot for Gag, ACTB, and LC3. All images are representative of 3 independent experiments. Significantly different values are indicated by asterisks ***P ≤ 0.001

In order to identify the genetic determinants that facilitate the autophagic recruitment and degradation of Gag, we performed immunoprecipitation assays and assessed the steady-state levels of three mutants of Gag. First, we used a G_2_A-Gag mutant, which cannot become myristoylated and thus, loses its ability to bind to membranes [47–51]. Second, since autophagy cargo is often poly-ubiquitinated, we introduced alanine substitutions at lysine residues in Gag predicted to become ubiquitinated (K_113_A, K_114_A, K_335_A, K_359_A, and K_418_A) generating the Ub-Gag mutant [44]. A third mutant (G_2_A/Ub-Gag) that lacks both the ability to become myristoylated and ubiquitinated was also generated. For this assay, HEK293T cells were transfected with the wild-type Gag-EGFP or the Gag-EGFP mutants and their interaction with the endogenous autophagy machinery was assessed by immunoprecipitation. Compared to wild-type Gag, LC3 interaction with the single mutants was significantly reduced, and almost completely abrogated for the double Gag mutant (Fig. 3A)—the relative interaction with LC3 was measured by densitometric analyses (Fig. 3A; bottom graph). Moreover, unlike wild type Gag, no significant fluctuations in the steady-state levels of the Gag mutants, particularly the double mutant, were observed after treatment with rapamycin for 12 h (Fig. 3B). Therefore, these findings indicate that not only Gag is specifically targeted by the autophagy machinery for autolysosomal clearance, but also that Gag ubiquitination and association with membranes are crucial for its autophagy-mediated recognition and degradation.

### Residues comprising positions 40 to 57 in the N-terminal domain of HIV-1 NL4-3 Nef are required to block the early stages of autophagy

**Fig. 4:**
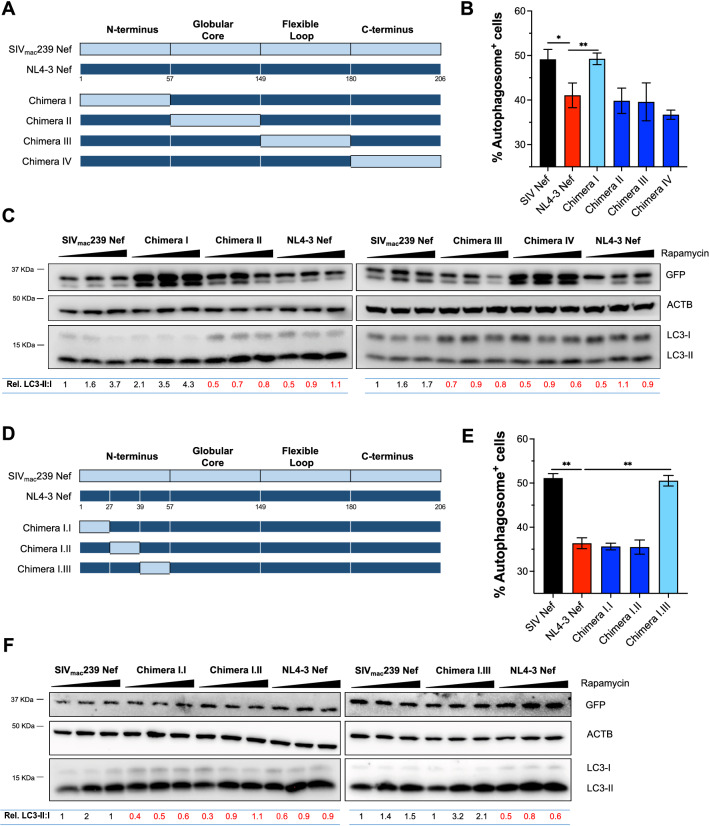
Residues 40–57 in the N-terminal domain of NL4-3 Nef are required to counteract autophagy initiation. **A**, **D** Schematic representation of the domains replaced for the generation HIV-1 NL4-3 and SIV_mac_239 Nef chimeras. **B**, **E** HEK293T cells were co-transfected with EGFP*-LC3B* and the different *nef* constructs: SIV_mac_239 *nef*, NL4-3 *nef* and the selected *nef* chimeras. 48 h post-transfection, cells were analyzed by flow cytometry for autophagosome-associated EGFP-LC3B. Data correspond to the mean and SEM of the percentage of EGFP^+^ cells from three independent experiments. **C**, **F** HEK293T cells were transfected with NL4-3 *nef*, SIV_mac_239 *nef* and the selected chimeras. 48 h later, cells were exposed for 4 h to increasing concentrations of rapamycin (0–4 μM). Next, cells were analyzed by western blot for the levels of GFP, LC3, and ACTB. Densitometric analyses were performed to determine the ratio of LC3-II over LC3-I relative to SIV_mac_239 *nef* with no rapamycin treatment. All images are representative of three independent experiments. Significantly different values are indicated by asterisks *P ≤ 0.05; **P ≤ 0.01

Unlike NL4-3 Nef, our previous work revealed that SIV_mac_239 Nef cannot counteract autophagy restriction [28]. We took advantage of this fact to generate chimeric Nef proteins where we swapped individual functional domains between these two proteins with the goal of performing a loss-of-function assay and reveal the specific residues within NL4-3 Nef responsible for autophagy antagonism. For this, we replaced the N-terminus, globular core, flexible loop and C-terminus in NL4-3 Nef by the ‘inactive’ domains from SIV_mac_239 Nef, generating the chimeras I, II, III and IV (Fig. 4A). In order to determine whether these chimeras were able to intersect with autophagy, we first evaluated the impact of the resulting proteins on autophagosome biogenesis by flow cytometry assays, employing the same EGFP-LC3 construct used in Fig. 2. Besides the chimeras, we included SIV_mac_239 Nef and NL4-3 Nef as negative and positive controls, respectively. The principle of these assays relies on the fact that EGFP-LC3 binds to autophagosomes during their elongation, making EGFP-LC3 resistant to saponin treatments. Hence, after washing cells with a saponin-based wash buffer, the EGFP signal detected correlates with autophagosome formation [28]. 48 h post-transfection, cells were treated with rapamycin for 4 h prior to flow cytometry processing. With the exception of chimera I-transfected cells, which had similar levels of autophagosome formation as those expressing SIV_mac_239 Nef, cells transfected with chimeras II, III and IV displayed low autophagosome biogenesis (Fig. 4B and Additional file 1: Fig. S1). To corroborate these observations, we next analyzed the effect of these chimeras on the relative levels of LC3 lipidation by western blot. All Nef constructs, including these chimeras, were cloned into the expression vector pCGCG, which harbors EGFP from an internal ribosomal entry site [52, 53]. This feature was especially useful for these assays, since in some instances the mutagenesis of the native proteins modified the epitope sequence where the anti-HIV/SIV Nef antibodies bind. Therefore, we analyzed transfection efficiency and the expression of our constructs by monitoring the levels of EGFP, as in previous studies [28, 52, 54–58]. Of note, expression of EGFP from this construct is cytosolic and, thus, does not interfere with our quantification of EGFP-LC3-containing autophagosomes, since it is washed away upon saponin treatment [28]. Consistent with the flow data, NL4-3 Nef, together with the chimeras II, III, and IV, reduced LC3 lipidation even upon stimulation with increasing concentrations of rapamycin (Fig. 4C). However, chimera I, as well as the negative control SIV_mac_239 Nef, showed significantly higher ratios of LC3-II:I and, thus, normal autophagy flux (Fig. 4C; see quantifications underneath the blots), which indicates that the capacity of NL4-3 Nef to block LC3 lipidation and formation of autophagosomes resides somewhere along the N-terminal domain of the protein.

In order to narrow down which particular region is responsible for this activity, we generated additional chimeric proteins replacing three portions within the N-terminal domain of NL4-3 Nef, as shown in Fig. 4D. Similar to our previous approach, we first tested the ability of chimeras I.I, I.II and I.III to impair autophagosome formation by measuring saponin resistant EGFP-LC3 using flow cytometry. Whereas chimeras I.I and I.II retained the full potential to limit autophagosome biogenesis, chimera I.III did not exhibit such effect on autophagy (Fig. 4E). Consistent with this, we also found that unlike the chimeras I.I and I.II, the chimeric protein I.III was not able to prevent LC3 lipidation upon autophagy activation by rapamycin (Fig. 4F). These observations indicate that the ability to inhibit the initiation of autophagy maps to a region between amino acids 40 and 57 in the N-terminal domain of NL4-3 Nef.

### Residues T_48_ and A_49_ in NL4-3 Nef are responsible for counteracting autophagy initiation

**Fig. 5:**
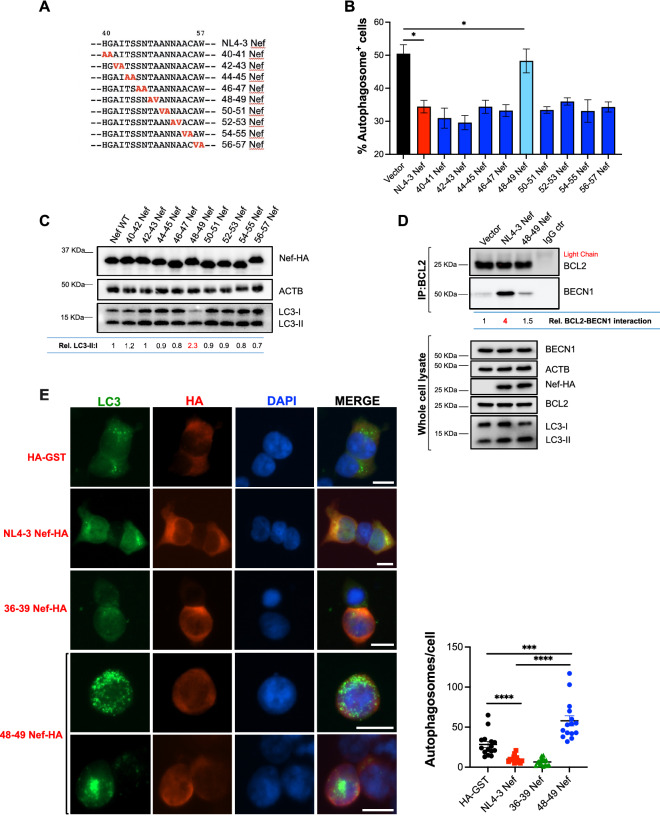
Nef uses residues 48–49 to prevent autophagosome biogenesis. **A** Alignment comprising residues 40–57 in NL4-3 Nef. Substitutions introduced in each mutant are indicated in red. **B** HEK293T cells were co-transfected with EGFP*-LC3B* and either an empty vector, NL4-3 *nef* or the selected *nef* mutants. 48 h post-transfection, cells were analyzed by flow cytometry for autophagosome-associated EGFP-LC3B. **C** HEK293T cells were transfected with NL4-3 *nef* or the selected mutants. 48 h later, cells were treated with rapamycin (4 μM) for 4 h and analyzed by western blot for HA, LC3, and ACTB. Densitometric analyses determine the ratio of LC3-II:I relative to NL4-3 *nef* are shown underneath the blots. **D** HEK293T cells were co-transfected with *BECN1* and either an empty vector, NL4-3 *nef* or 48–49 NL4-3 *nef*. 48 h later, cells were harvested and BCL2 was immunoprecipitated. The pulldown fraction was examined for BCL2 and BECN1. Lysates were also analyzed by western blot for BECN1, HA, BCL2, LC3, and ACTB. Densitometric analyses indicate the relative BCL2-BECN1 interaction. **E** HEK293T cells stably expressing EGFP-LC3 were transfected with an empty vector, NL4-3 *nef*, a Nef mutant harboring alanine substitutions at the residues responsible for blocking autophagy maturation (36–39 Nef) or 48–49 NL4-3 *nef*. 48 h later, cells were treated with DMSO or rapamycin (4 μM) for 4 h prior to microscopy visualization of EGFP-LC3-coated autophagosomes. Graph: number of autophagosomes per cell from 15 randomly selected cells. Scale bar: 10 μm. Images are representative of three independent experiments. Significantly different values are indicated by asterisks *P ≤ 0.05; ***P ≤ 0.001; ****P ≤ 0.0001

To identify the specific residues in Nef required to intersect with the early stages of autophagy, we generated 9 pair-wise alanine-scanning mutants by site-directed mutagenesis comprising positions 40 to 57 (Fig. 5A). Of note, positions that naturally harbor alanine residues were replaced by valine. Next, the mutants were tested for their effect on autophagy initiation. For convenience, this panel of mutants along with wild type NL4-3 Nef was cloned into the expression vector pCI and were tagged with HA to facilitate western blot analyses and microscopy studies. For these assays, HEK293T cells were transfected with each of the 9 mutants, using wild-type NL4-3 Nef and an empty vector as positive and negative controls, respectively. As for Fig. 4, their impact on autophagosome biogenesis was assessed first by flow cytometry. All mutants except construct 48–49 Nef (which harbors T_48_A and A_49_V substitutions) were successful at preventing autophagosome formation, which is reflected by a significant reduction in the percentage of autophagosome^+^ cells (Fig. 5B). Consistent with these findings, mutation of residues 48–49 abrogated Nef’s ability to limit autophagy flux, since when cells expressing this mutant were treated with rapamycin a rapid LC3-I-to-LC3-II conversion was observed (Fig. 5C). We previously demonstrated that HIV-1 NL4-3 Nef prevents early stages of autophagy by enhancing an association between BECN1 (a key protein in autophagy initiation) and its natural inhibitor BCL2 [28]. Hence, we sought to investigate the effect of mutations at positions 48–49 in Nef in the BECN1-BCL2 binding. For this, HEK293T cells were transfected with an empty vector (pCI), NL4-3 Nef or the 48–49 Nef mutant. 48 h later, cells were harvested and BCL2 was immunoprecipitated using a BCL2-specific antibody. The pulldown fraction was next analyzed for the presence of BCL2 and BECN1, as previously described [28]. In agreement with our previous findings, wild type NL4-3 Nef, but not the 48–49 Nef mutant, enhanced the association between BECN1 and BCL2 (Fig. 5D). Hence, these observations demonstrate that the ability of Nef to intersect with autophagy initiation through the BCL2-mediated sequestration of BECN1 maps to residues 48–49 in Nef.

Previous studies have reported that the ability of Nef to impair autophagosome-lysosome fusion maps to residues 36–39, which resemble the domain that Rubicon uses to block autophagy maturation [28, 31]. To confirm that Nef’s abilities to intersect with autophagy initiation and maturation are genetically separable, we assessed autophagosome biogenesis by fluorescence microscopy. For this, HEK293T cells stably expressing EGFP-LC3 were transfected with HA-GST (as an irrelevant protein), NL4-3 Nef, a Nef mutant harboring alanine substitutions at positions 36–39, or the 48–49 Nef mutant. 48 h later, cells were exposed to rapamycin (4 μM) for 4 h, and autophagosome formation was monitored by LC3 puncta. Consistent with our previous findings [28], wild type Nef significantly reduced autophagosome biogenesis. A similar phenotype is observed with the 36–39 Nef mutant, which is unable to block autophagy maturation. This is expected, since this mutant is still able to intersect with autophagy initiation [28]. By contrast, cells expressing the 48–49 Nef mutant displayed high levels of autophagosomes, reflecting its inability to prevent their generation. However, the level of autophagosomes was higher than that of the HA-GST control, supporting the notion that this mutant retains the ability to block autophagosome–lysosome fusion, causing in turn an accumulation of autophagosomes (Fig. 5E). Quantification of autophagosomes from 15 randomly selected cells for each experimental condition further confirms these observations (Fig. 5E; graph). Hence, these findings demonstrate that the ability of Nef to intersect with autophagy initiation and maturation is genetically separable.

### The Nef-mediated block in autophagy initiation prevents Gag redistribution to autophagosomes, consequently increasing virion production

**Fig. 6:**
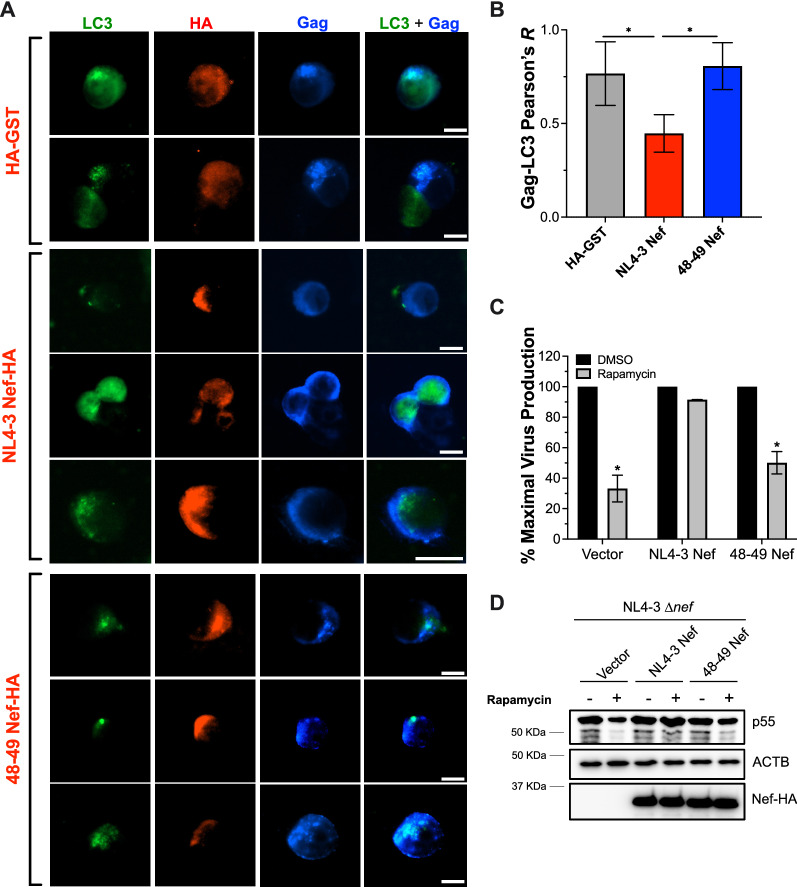
Nef uses residues 48–49 to prevent Gag redistribution to autophagosomes, restoring Gag and virion levels. **A** HEK293T cells stably expressing EGFP-LC3 were co-transfected with NL4-3 Δ*nef* and either HA-GST (irrelevant protein), NL4-3 *nef*, or 48–49 NL4-3 *nef*. 48 h later, cells were stimulated with rapamycin (4 μM) for 4 h and autophagosome formation was examined by fluorescence microscopy. **B** The Pearson’s correlation coefficient for the Gag-LC3 co-localization was analyzed from three biological replicates. Scale bar: 10 μm. Images are representative of three independent experiments. **C** HEK293T were co-transfected with NL4-3 Δ*nef* and either an empty vector, NL4-3 *nef*, or 48–49 NL4-3 *nef*. 24 h later, cells were treated with DMSO or rapamycin (4 μM) for 12 h. The percentage of maximal virus release was determined by p24 ELISA of the culture supernatants. **D** Lysates were also analyzed by western blot for Gag, HA and ACTB. Significantly different values are indicated by asterisks *P ≤ 0.05

To determine the relevance of Nef’s effects on counteracting the early stages of autophagy in Gag levels, and thus, virus replication, we assessed the subcellular distribution of Gag in the presence of Nef and the 48–49 Nef mutant. For this, HEK293T cells stably expressing EGFP-LC3 were co-transfected with NL4-3 Δ*nef* and either HA-tagged GST, NL4-3 Nef or 48–49 Nef. 48 h later, cells were treated with rapamycin (4 μM) for 4 h to trigger autophagy, and the co-localization of Gag and LC3 was analyzed by fluorescence microscopy. The degree of Gag-LC3 co-localization was determined by calculating the Pearson’s correlation coefficient. As expected, Gag was mainly distributed at the plasma membrane in the presence of wild type Nef, while it largely localized in LC3-coated autophagosomes in cells expressing HA-GST or the 48–49 Nef mutant (Fig. 6A, B). These findings are consistent with our replication assays in Fig. 1, where Nef expression is associated with higher Gag levels, and further support the idea that by impairing autophagosome formation, Nef prevents Gag from being targeted for autophagy elimination. This hypothesis was verified through particle rescue assays. Here, HEK293T cells were co-transfected with NL4-3 Δ*nef* and either pCI, NL4-3 Nef or the 48–49 Nef constructs. 24 h post-transfection, cells were washed, and the culture media was supplemented with rapamycin (4 μM) or DMSO for 12 h. The percentage of maximal virus production was calculated relative to the levels of virions detected in the presence of DMSO for each transfection condition. Whereas the presence of Nef rescued virion production in the presence of rapamycin, the 48–49 Nef mutant failed at doing so (Fig. 6C). In fact, when analyzing the cell lysates, Gag levels were only fully restored by wild type Nef (Fig. 6D). However, despite its inability to rescue virion release to the levels of wild type Nef under rapamycin treatment, cells transfected with the 48–49 Nef mutant afforded higher Gag expression (Fig. 6D) and virion production than those transfected with the pCI vector (Fig. 6C; 50% versus 33% of maximal virus release, respectively), suggesting that the ability of Nef to restore Gag levels, and consequently virion release, requires Nef-mediated block on both autophagy initiation and maturation.

### The ability of Nef to block autophagy initiation is genetically separable from other functional roles of Nef

**Fig. 7:**
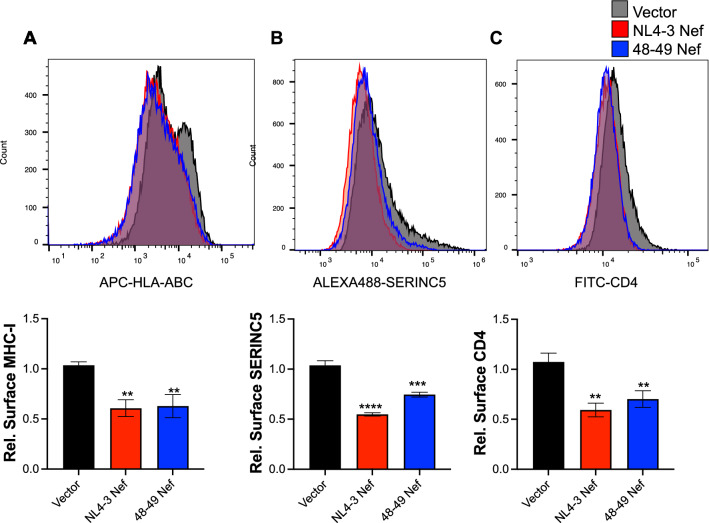
The ability of Nef to impair autophagy initiation is genetically separable from other functional roles of the protein. **A**, **B** HEK293T cells were co-transfected with SERINC5-HA and either an empty vector, NL4-3 *nef* or 48–49 NL4-3 *nef*. 48 h later cells were analyzed by flow cytometry to measure the surface levels of MHC-I (**A**), and SERINC5 relative to the empty vector control (**B**). **C** HeLa TZM-bl cells were transfected with an empty vector, NL4-3 *nef* or 48–49 NL4-3 *nef*. 48 h later, cells were analyzed by flow cytometry for their surface levels of CD4 relative to the empty vector control. Data correspond to the mean and SEM of three biological replicates. Histograms are representative of three independent experiments. Significantly different values are indicated by asterisks **P ≤ 0.01; ***P ≤ 0.001; ****P ≤ 0.0001

To investigate whether mutations at residues T_48_ and A_49_ only affect the ability to counteract autophagy initiation or if they also impact other functional roles of Nef—probably due to destabilization of the protein—we compared the 48–49 Nef mutant with wild type NL4-3 Nef for its ability to down-regulate the membrane proteins MHC-I, SERINC5 and CD4, well-known functions of Nef that afford immune evasion [24–26]. HEK293T cells were used for the SERINC5 and MHC-I assays. Due to the low levels of endogenous SERINC5 in this cell line, cells were transfected with an empty vector or an expression vector encoding SERINC5. In addition, constructs encoding NL4-3 Nef, 48–49 Nef, or the empty pCI vector were included in these transfections. 48 h later, the surface levels of MHC-I and SERINC5 were examined by flow cytometry. Although we detected minor differences compared to wild type Nef in the ability to down-regulate SERINC5, the 48–49 Nef mutant still significantly reduced the surface levels of both SERINC5 and MHC-I (Fig. 7A, B). HeLa TZM-bl cells, which are engineered to endogenously express CD4 and CCR5 [59], were used for the CD4 down-regulation assays, since HEK293T cells do not express CD4 endogenously. Cells were transfected with NL4-3 Nef, 48–49 Nef, or the empty pCI vector and 48 h later they were analyzed for the surface levels of CD4 by flow cytometry. Similar to our observations on MHC-I and SERINC5, 48–49 Nef potently down-regulated CD4 (Fig. 7C). Therefore, the ability to block early stages of autophagy maps to residues T_48_ and A_49_ in Nef and this activity is genetically separable from other major functions of this protein.

### HIV-1 transmitted/founder viruses conserve the ability to counteract autophagy

**Fig. 8:**
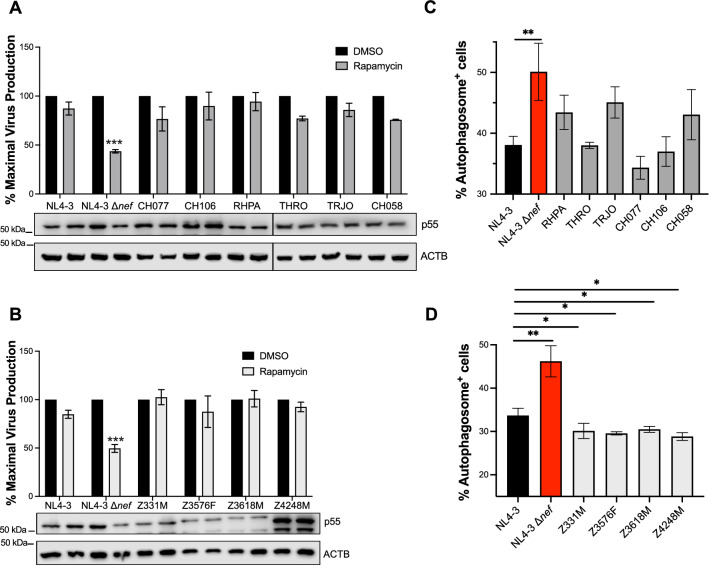
HIV-1 transmitted/founder (T/F) viruses from subtypes B and C conserve the ability to antagonize autophagy. **A**, **B** HEK293T cells were transfected with the proviral DNA of HIV-1 NL4-3, HIV-1 NL4-3 Δ*nef* or the selected T/F clones that belong to the pandemic HIV-1 subtype B (**A**) or subtype C (**B**). 24 h later, the cell medium was replaced and supplemented with rapamycin (6.5 μM) or DMSO. 18 h later, the percentage of maximal virus production was measured by the accumulation of HIV p24 in the culture supernatant relative to the DMSO treatment. Bottom blots: Cell lysates were also analyzed by western blot for p55 and ACTB. In each case, the percentage of maximal virus production is indicated as the mean and SEM from 3 independent biological replicates. **C**, **D** HEK293T cells were co-transfected with EGFP*-LC3B* and the proviral DNA of HIV-1 NL4-3, HIV-1 NL4-3Δ*nef* or the selected T/F clones that belong to the pandemic HIV-1 subtype B (**C**) and subtype C (**D**). 48 h post-transfection, cells were treated with DMSO or rapamycin and subsequently analyzed by flow cytometry for autophagosome-associated EGFP-LC3. Data correspond to the mean and SEM from three independent replicates. Significantly different values are indicated by asterisks *P ≤ 0.05; **P ≤ 0.01; ***P ≤ 0.001

Our previous work pointed to the evolutionary relevance of Nef’s ability to counteract the initiation of autophagy [28]. Particularly, we found that this activity is conserved among pandemic clades of HIV-1 but missing in non-pandemic clades as well as HIV-2 isolates, suggesting a potential role for autophagy antagonism in the successful global spread of HIV-1 [28]. If this is the case, resistance to autophagy restriction should be a conserved phenotypic trait among pandemic HIV-1 transmitted/founder (T/F) viruses. T/F viruses play an important role in mucosal transmission where, due to selective forces, only variants with high resistance to innate immune barriers are capable of infecting the new host and establish a chronic infection. Hence, studies on T/F viruses are of great interest, since they help uncover the immune blockades that need to be circumvented in order to establish de novo infections [32, 34, 35]. Based on this knowledge, we evaluated the endurance of T/F viruses to rapamycin treatments and their capacity to prevent formation of autophagosomes. We selected a panel of ten different T/F clones that belong to the pandemic subtypes B and C. For this study, HEK293T cells were transfected with the proviral DNA of these clones, wild-type NL4-3 or NL4-3 Δ*nef*, which were used as positive and negative controls, respectively. Autophagy was induced by treating cells with 6.5 μM rapamycin for 18 h. Next, cell lysates were collected and analyzed by western blot, and the culture supernatants were used to measure virion production by p24 antigen-capture ELISA. Remarkably, all T/F viruses showed little reduction on both viral release and intracellular levels of HIV Gag upon rapamycin treatment, in contrast to the great impact observed on the autophagy-sensitive NL4-3 Δ*nef* (Fig. 8A, B), indicating the resistance of this T/F panel to autophagy restriction. In agreement with this finding, most viruses reduced autophagosome biogenesis (Fig. 8C, D), and this was especially obvious for the T/F isolates that belong to subtype C (Fig. 8D), which afforded a higher inhibitory effect on blocking autophagosome formation than wild type NL4-3. Hence, altogether these results indicate that pandemic T/F viruses intersect with the generation of autophagosome structures, and therefore, counteract autophagy-mediated restriction. Importantly, resistance to autophagy was observed in all T/F primary isolates tested and suggests that autophagy antagonism is critical for HIV-1 infectivity and transmission.

## Discussion

Autophagy is a degradative and highly conserved pathway that is important for cellular homeostasis. In addition, autophagy serves as a potent mechanism of defense against viruses and other intracellular pathogens [20, 60]. Not only autophagy targets virions and viral components for degradation, but also aids in the activation of innate and adaptive responses against these parasites. Hence, the pharmacological manipulation of this pathway represents an attractive approach for the successful elimination of viruses such as HIV. However, in order to consider the exploitation of autophagy in therapeutic strategies against this virus, a complete understanding of the interplay between HIV and the autophagy machinery is necessary. In this regard, we recently reported that the pharmacological activation of autophagy successfully restricts HIV replication. Specifically, autophagy causes a significant reduction in Gag levels, which consequently leads to a defect in virion production. However, HIV has evolved the virulence factor Nef to counteract such effect [28]. In line with those studies, here we demonstrate that this autophagy-mediated restriction is magnified when treatments with autophagy-activating drugs, such as rapamycin, are sustained over time, affecting sequential rounds of viral replication. These observations were reproduced in both CD4^+^ T cell lines and primary CD4^+^ T cells. In both scenarios, autophagy activation caused a striking reduction in the emergence of HIV Gag and in turn in virus replication. As anticipated, these effects were especially evident in the absence of HIV Nef. Rapamycin is an FDA-approved immunosuppressive drug commonly used after organ transplantation [61]. It has also been recommended for HIV^+^ individuals on latency reversing agents to reduce the cytokine-associated cytotoxicity caused by these drugs [62]. Hence, in these settings, the probability of HIV to encounter cells that are maximally induced for autophagy is high, which speaks of the physiological relevance of our findings. However, the effects of sustained autophagy activation by rapamycin on the *nef*-deleted virus were more striking in Jurkat cells than in primary cells. As reported by us and others, the activation of naïve CD4^+^ T cells potently triggers autophagy [28, 63–70]. As a consequence of this, these cells are better equipped to fight an infection than the Jurkat cells. Hence, it is likely that any incoming virus is already being impacted in this *milieu*. Moreover, given these high autophagy levels, rapamycin may not be able to further increase this activity, which would explain why the differences between the rapamycin and mock treatments are less dramatic in the primary cells. Nevertheless, these data still support the potential of the pharmacological activation of autophagy to limit HIV replication.

Our previous work pointed that the restrictive effect that autophagy imposes on HIV is due to a defect in Gag, most likely by targeting this protein for autolysosomal degradation [28]. Here we confirm this hypothesis. First, inhibition of lysosomal function but not proteasomal function rescues Gag levels. Second, Gag physically interacts with LC3, and this interaction might also occur with the unlipidated LC3 variant—which may explain why we detect an association between Gag and LC3 even in the presence of Nef. Third, Gag expression and recruitment by the autophagy machinery is greatly influenced by SQSTM1. Finally, Gag co-localizes with LC3-coated autophagosomes under conditions of autophagy activation. However, since Gag is naturally associated with cellular membranes by virtue of a myristoyl group, its presence in autophagosomes might be coincidental. This possibility was ruled out by assessing whether other membrane-associated HIV proteins could similarly be impacted by autophagy. Our proteasomal/lysosomal assays and immunoprecipitations confirmed that unlike Gag, gp120 and Nef are not affected by autophagy activation nor associate with LC3. Although these observations corroborate that Gag’s presence in autophagosomes is specific, our mapping assays revealed that the ability of the autophagy machinery to target Gag relies on the capacity of this protein to bind to membranes and to become ubiquitinated. Actually, most autophagic cargo is ubiquitinated. The finding that Gag ubiquitination is necessary for its autophagy-mediated clearance suggests that this post-translational modification enables Gag recognition by the ubiquitin associated domain (UBA) of autophagic receptors. SQSTM1 is the main autophagy receptor that recruits ubiquitinated cargo [12, 13, 71]. Hence, the interaction between Gag and SQSTM1 would be in line with this mechanism. The fact that Gag also needs to associate with membranes for its successful elimination in autolysosomes seems unrelated. However, the process that mediates the ubiquitination of Gag is highly dependent on Gag association with membranes [72]. This phenomenon would in turn explain why the G_2_A-Gag mutant (unable to bind to membranes), the Ub-Gag mutant (unable to become ubiquitinated) but particularly the G_2_A/Ub-Gag mutant fail at being recognized by the autophagy machinery (evidenced by a reduction in LC3 association), and consequently are resistant to autophagic degradation. The next logical step to further verify these findings would involve engineering a NL4-3 clone harboring mutations in Gag at the myristoyl and ubiquitination sites. However, both post-translational modifications are fundamental for Gag’s role in virion assembly and budding [73, 74]. Therefore, such mutant would be severely compromised. Taken together, our data indicate that due to the central role of Gag in the formation of viral particles, the autophagy-mediated elimination of this protein impairs the steps of virion assembly and release and thus, the overall replicative capacity of HIV.

The identification of the genetic determinants in Gag that render this protein vulnerable to autophagy restriction only provide us with a partial picture of the HIV-autophagy interplay. In our previous studies, we characterized the mechanism by which Nef impairs autophagosome formation, but we did not map the domains in Nef required for this activity. To address this, we generated chimeric Nef proteins between active (NL4-3 Nef) and ‘inactive’ (SIV_mac_239 Nef) Nef alleles [28] in which the individual functional domains of the protein were replaced. The resulting chimeras were tested for their ability to intersect autophagy by measuring autophagosome formation (flow cytometry) and autophagy flux (western blot). These initial assays pointed to a region within the N-terminus of NL4-3 Nef required to intersect with the early stages of autophagy. We next introduced pair-wise amino acid substitutions in this region and tested the resulting mutants as detailed above. Our results show that amino acids T_48_ and A_49_ in NL4-3 Nef are required to block autophagosome biogenesis. In particular, specific point mutations on these residues abrogated Nef’s ability to enhance the BCL2-BECN1 interaction, and therefore, its ability to impair LC3 lipidation and formation of autophagosomes. As a consequence of this, a Nef mutant harboring substitutions at residues 48–49 was unable to prevent Gag redistribution to autophagosomes and, thus, restore Gag levels and virion production under conditions of rapamycin-induced autophagy. However, other relevant functions of Nef such as the down-regulation of MHC-I, SERINC5 or CD4 were not affected. Although mutation at residues 48–49 had a minor impact on SERINC5 down-regulation, the mutant protein was still able to significantly reduce the surface levels of SERINC5. Interestingly, previous studies on the Nef-autophagy interplay showed that residues D_36_LEK_39_, also found in the N-terminal domain of the protein, were required for the Nef-mediated impairment of autophagy maturation [31]. We previously confirmed the findings of that study by introducing alanine substitutions in that motif, which significantly affected Nef’s ability to block maturation. However, these replacements had no effect on the ability of Nef to inhibit autophagy initiation [28]. Similarly, while the 48–49 Nef mutant lost the capacity to block autophagy initiation, our microscopy studies show that it retains the ability to intersect with autophagy maturation. Hence, our results demonstrate that despite being ten amino acids apart, the determinants that govern Nef’s actions over autophagy initiation and maturation are in fact genetically separable. In future studies, we will investigate how mutations that specifically impair Nef’s ability to block autophagy initiation, maturation or both impact viral infectivity and fitness.

Although dispensable for replication in vitro, Nef is essential for infectivity in vivo. Nef achieves this by affording immune evasion through multiple mechanisms [25, 26, 75–77], and autophagy antagonism may be part of it. Consistent with this notion, our previous work pointed to a potential role for the Nef-mediated counteraction of autophagy in transmission and spread, since this activity was found highly conserved among Nef alleles of the most widely distributed pandemic clades of HIV-1 [28]. In fact, a recent publication demonstrated that treatments with different autophagy-enhancing drugs potently reduce viral transmission in mucosal ex vivo models [78]. Since T/F viruses are responsible for successful transmission events among individuals, we evaluated the susceptibility of a panel of pandemic HIV-1 T/F viruses to autophagy restriction. For this, we selected isolates from the HIV-1 subtypes B and C, which together represent more than 60% of all current HIV-1 infections worldwide [79, 80]. Strikingly, all the T/F clones tested, especially those belonging to subtype C, displayed high resistance to autophagy restriction as well as the capacity to prevent autophagosome formation. Future work will expand the T/F virus library and study the susceptibility of their Gag proteins to autophagy restriction as well as the role of their *nef* genes in autophagy antagonism. Overall, our findings suggest that T/F viruses circumvent autophagy, and that this activity could be partially responsible for their distinctive viral fitness and their capacity to overcome mucosal immune barriers. Hence, these observations further support the idea that autophagy counteraction plays an important role in HIV-1 transmission.

## Conclusions

In this report, we have demonstrated that autophagy antagonism is important for virus replication and have identified the genetic determinants that drive the mutual antagonism between HIV and autophagy. First, we found that autophagy restriction is accomplished through the ubiquitin-dependent recognition and autolysosomal degradation of the virus protein Gag. Second, we uncovered that HIV-1 Nef-mediated inhibition of autophagy initiation requires residues T_48_ and A_49_. Finally, our studies with HIV-1 transmitted/founder viruses indicate that autophagy antagonism might be crucial for mucosal transmission. Therefore, these findings could open new avenues for the design of approaches aimed at rendering the virus susceptible to autophagy elimination or even novel PreP regimens aimed at intersecting mucosal transmission.

## Methods

### Plasmids and DNA constructs

The following full-length proviral constructs were obtained through the NIH AIDS Reagent Program, Division of AIDS, NIAID, NIH. Wild-type HIV-1 NL4-3 (pNL4-3, #114) and NL4-3 Δ*nef* (pNL4-3 ΔNef, #12755) were obtained from Drs. Malcolm Martin and Olivier Schwartz, respectively [81, 82]. The proviral constructs for subtype B transmitted/founders clones CH077 (pCH077.t/2627, #11742), CH106 (pCH106.c/2633, #11743), RHPA (pRHPA.c/2635, #11744), THRO (pTHRO.c/2626, #11745), TRJO (pTRJO.c/2851, #11747), and CH058 (pCH058.c/2960, #11856) were obtained from Dr. John Kappes and Dr. Christina Ochsenbauer. The subtype C transmitted/founder clones Z331M (pZ331M, #13248), Z3576 (pZ3576F, #13256), Z3618 (pZ3618M, #13262), and Z4248 (pZ4248M, #13277) were obtained from Dr. Eric Hunter [34, 36, 83]. HIV-1 viruses based on these plasmids were generated by transient transfection in HEK293T cells, as previously described [28, 52, 55].

The expression vector pCGCG (a gift from Dr. Jacek Skowronski [Case Western Reserve University, Cleveland, OH]) harbors EGFP from an internal ribosomal entry site and was used to clone HIV-1 NL4-3 Nef and SIV_mac_ 239 Nef [28, 52, 84] using the XbaI and MluI unique restriction sites. The four chimeric proteins between NL4-3 Nef and SIV_mac_239 Nef (I, II, III, IV) were obtained by separately replacing four regions (N-terminus, globular core, flexible loop and C-terminus) in NL4-3 Nef with the corresponding domains in SIV_mac_239 Nef using overlapping PCR. The subsequent three chimeric proteins between NL4-3 Nef and SIV_mac_239 Nef (I.I, I.II, I.III) were obtained by replacing three regions in NL4-3 Nef N-terminus (I.I: residues 1–27, I.II: residues 28–39, II.III: residues 40–57) by the corresponding regions in SIV_mac_239 Nef using overlapping PCR. In addition, HA-tagged NL4-3 Nef was obtained from Addgene: pCI-NL4-3 *nef*-HA-WT (#24162, Dr. Warner Greene’s lab). The different pairwise amino acid mutants of NL4-3 Nef (NL4-3 Nef_40–41_, Nef_42–43_, Nef_44–45_, Nef_46–47_, Nef_48–49_, Nef_50–51_, Nef_52–53_, Nef_54–55_, Nef_56–57_) were obtained by site-directed mutagenesis of pCI-NL4-3 *nef*-HA-WT using quickchange PCR. Alanine residues were substituted by valine whereas any other amino acid was replaced by alanine.

The expression constructs pC3-*EGFP*-*LC3B* (#11546, Dr. Karla Kirkegaard’s lab) and pcDNA4-BECN1-Flag (#24388, Dr. Qing Zhong’s lab) were obtained through Addgene. HIV-1 Gag (p*Gag*-*EGFP*) was obtained through the NIH AIDS Reagent Program, Division of AIDS, NIAID, NIH from Dr. Marilyn Resh [85, 86]. Gag mutants G_2_A-Gag, Ub-Gag and the double mutant G_2_A/Ub-Gag were generated by quickchange site-directed mutagenesis of p*Gag*-GFP, as described before [44]. Human *SERINC5* was synthesized by IDT (Integrated DNA Technologies, Coralville, IA) as a mini-gene into a cloning vector. Subsequently, *SERINC5* was subcloned into the pcDNA5 expression vector using the unique restriction sites Kpn I and BamH I. An HA tag was introduced in the C-terminal domain to facilitate protein detection.

### Transfections

6 × 10^5^ HEK293T (American Type Culture Collection [ATCC], CRL-11268) cells were plated 24 h before transfection. Cells were transfected with 2000 ng of each expression construct using GenJet in vitro DNA transfection reagent (SignaGen Laboratories, SL100488), following the manufacturer’s suggestions. For the SQSTM1 depletion studies, 8 × 10^5^ HEK293T cells were transfected with 100 nM of SignalSilence siRNA II specific for SQSTM1 (Cell Signaling, #6399) using Lipofectamine 3000 (ThermoFisher Scientific, #L3000001), following the manufacturer’s instructions. 24 h later, the medium was replaced, and cells were transfected once again with 2000 ng of NL4-3 Δ*nef* proviral DNA using GenJet in vitro transfection reagent. For every transfection, cell viability was monitored to evaluate potential cellular toxicity. No evidence of toxicity was observed since viability usually ranged between 90 and 100%.

### Infections

10^6^ Jurkat CD4^+^ T cells (ATCC, TIB-152) or 10^6^ primary CD4^+^ T cells (Zen-Bio, Inc., SER-PBMCD4 + TH-N-F) were infected with 100 ng of p24 equivalents of HIV-1 NL4-3 or NL4-3 Δ*nef* by spinoculation for 3 h at 37 °C. Prior to infection, primary naïve CD4^+^ T cells were activated using 25 μL anti-CD3/CD28 beads (Invitrogen, #111.31D), 1 μg/mL of IL-4 (Peprotech, Rocky Hill, NJ, #500-P24), 2 μg/mL IL-12 (Peprotech, #500-P154G), 1 ng/mL TGF-B (Peprotech, #100-21) and expanded for 3 days in RPMI medium (ThermoFisher Scientific, #11875-119) supplemented with 10% of fetal bovine serum (ThermoFisher Scientific, #26140-079) and 30 IU/mL of IL-2 (NIH AIDS Reagent Program, Division of AIDS, NIAID, NIH; #136). After infection, cells were washed and re-suspended in 4 mL of RPMI medium supplemented with 10% of fetal bovine serum and 30 IU/mL of IL-2 (IL-2 was added only for primary cells). Next, cells were either treated with DMSO or rapamycin (Sigma-Aldrich, #R8781) at 6.5 μM, which was sustained until the end of the experiment. Samples were analyzed at 6, 24, 48 and 72 h post-infection. For the 3-MA assays, cells were washed 24 h post-infection and supplemented with DMSO, rapamycin (6.5 μM) or a combination of rapamycin (6.5 μM) and 3-MA (3 mM) for 6 h. Cell lysates as well as supernatants were subjected to western blot and p24 antigen-capture ELISA, respectively (Advanced Biolabs, 5421 and 5436), respectively.

### Virus release assays

6 × 10^5^ HEK293T cells were transiently transfected with 2000 ng of full-length provirus of HIV-1 clones NL4-3, NL4-3 Δ*nef* or transmitted/founder clones CH077 (pCH077.t/2627), CH106 (pCH106.c/2633), RHPA (pRHPA.c/2635), THRO (pTHRO.c/2626), TRJO (pTRJO.c/2851), CH058 (pCH058.c/2960), Z331M (pZ331M), Z3576 (pZ3576F), 3618 (pZ3618M), and Z4248 (pZ4248M). Thirty-six hours post-transfection, the cell medium was replaced, and rapamycin was added at 6.5 μM. Forty-eight hours post-transfection, the culture supernatants were collected and analyzed by p24 antigen-capture ELISA (Advanced Biolabs, 5421 and 5436), as previously described [28, 44, 52, 53, 55, 87]. In addition, cells were washed, lysed, and the whole cell lysates were analyzed by western blotting.

Similar assays were performed for the virus release rescue experiments. In this case, cells were co-transfected with 2000 ng of the proviral DNA of NL4-3 Δ*nef* and 3000 ng of either the empty vector pCI, pCI-NL4-3-Nef or pCI-NL4-3-Nef_48–49_. Thirty-six hours post-transfection, the cell medium was replaced, and rapamycin was added at 4 μM.

### Gag degradation assays

6 × 10^5^ HEK293T cells were transfected with 2000 ng of full-length proviral DNA of HIV-1 NL4-3 Δ*nef*; p*Gag*-EGFP or p*Gag*-EGFP mutants. Twenty-four hours post-transfection, the cell medium was replaced and rapamycin was added at 4 μM for 12 h. Additional treatments included (i) rapamycin in combination with chloroquine (60 μM; Sigma-Aldrich, #C6628-256), and (ii) rapamycin plus ALLN (25 μM; EMD Millipore, #208750-5MG) for 12 h. Cells were then washed, lysed and harvested for their analysis by western blotting.

### Western blotting

HEK293T, Jurkat and primary CD4^+^ T cells were washed using DPBS and harvested in lysis IP buffer (Thermo Scientific, #87787) supplemented with protease inhibitors (Roche, #04693116001) and phosphatase inhibitor cocktails 2 and 3 (Sigma-Aldrich, #P5726 and #P0044). Cell lysates were incubated on ice for 1 h and then centrifuged at 16,000×*g* for 8 min to pellet down cell debris. The supernatant was collected and mixed 1:1 with 2× SDS sample buffer (Sigma-Aldrich, #S3401) before being boiled for 5 min. Proteins were then separated by electrophoresis on SDS-PAGE polyacrylamide gels (8–12%) and transferred to a polyvinylidene difluoride (PVDF) membrane (BioRad, #1620177) using a Trans-Blot SD (BioRad, #1703940) or Trans-Blot Turbo (BioRad, #1704150) transfer systems. After protein transfer, the PVDF membranes were incubated at room temperature for 1 h with blocking buffer (BioRad, #1706404). Membranes were then incubated with the respective primary antibodies overnight at 4 °C (antibody sources and dilutions are detailed in Table 1). Next, membranes were washed 3 times in PBS-tween (Sigma-Aldrich, #P3563) for 15 min at room temperature prior to their incubation for 1 h with the secondary antibody (antibody sources and dilutions are detailed in Table 1). After the incubation with the secondary antibodies, the membranes were washed 3 additional times and developed using SuperSignal West Femto maximum-sensitivity substrate (Pierce, 34095). Finally, imaging was carried out using a Li-Cor Odyssey Fc Imager 2800 (Li-Cor, Lincoln, NE) and Chemidoc™ Imaging System (BioRad, 12003153). Densitometric analyses were performed using Image Studio (Li-Cor) and Image Lab (BioRad) softwares.

**Table 1:** Antibody sources and conditions

Protein or tag	Antibody	Dilution	Source
ACTB/β-actin	Mouse monoclonal (C4) to ACTB/β-actin	1:1000	Sigma-Aldrich, MAB1501
BCL2	Mouse monoclonal (124) to BCL2	1:1000	Cell Signaling Technology, 15071S
BECN1	Rabbit monoclonal (D40C5) to BECN1	1:1000	Cell Signaling Technology, 3495S
CD4	Mouse monoclonal (RPA-T4) to CD4 (FITC-conjugated)	1:200	BD biosciences, 561005
GFP	Mouse monoclonal (4B10B2)	1:1000	Sigma-Aldrich, SAB5300167
LC3	Rabbit polyclonal and Rabbit monoclonal (D11) to LC3B	1:1000	Cell Signaling Technology, 2775S and 3868S
HA	Mouse monoclonal (16B12) to HA Rabbit polyclonal to HA	1:1000 1:200 (microscopy) 1:100	Covance, MMS-101R Abcam, ab137838
HIV Nef	Mouse monoclonal (2H12) to HIV-1 Nef	1:1000	ThermoFisher Scientific, MA1-71505
HIV-1 Gag p55/p24	Mouse monoclonal (183-H12-5C) to HIV p24	1:1000 1:200 (microscopy)	NIH AIDS Reagent Program, 3537
HIV-1 gp120	Goat polyclonal to HIV-1 gp120	1:1000	Abcam, ab21179
HLA-ABC	Mouse monoclonal (G46-2.6) to human HLA-ABC (APC-conjugated)	1:200	BD Biosciences 562006
SERINC5	Rabbit polyclonal to SERINC5	1:200	Sigma HPA037898
SQSTM1/p62	Mouse monoclonal to SQSTM1/p62	1:1000	Abcam, ab56416
SIV Nef	Mouse monoclonal (17.2) to SIV Nef	1:1000	NIH AIDS Reagent Program, 2659
Mouse IgG	Goat polyclonal (HRP-conjugated)	1:2000	Pierce, 31430
Mouse IgG1	Goat polyclonal (Alexa-568 conjugated)	1:500	ThermoFisher Scientific, A21134
Mouse IgG1	Goat polyclonal (Alexa-350 conjugated)	1:200	ThermoFisher Scientific, A21120
Rabbit IgG	Goat polyclonal (HRP-conjugated)	1:2000	Abcam, ab97051
Rabbit IgG	Donkey polyclonal (HRP-conjugated)	1:2000	Abcam, ab16284
Rabbit IgG1	Goat polyclonal (Alexa-488 conjugated)	1:200	ThermoFisher Scientific, A11008
Rabbit IgG	Goat polyclonal (Alexa-568 conjugated)	1:200	ThermoFisher Scientific, A11011
Goat IgG	Donkey polyclonal (HRP-conjugated)	1:2000	Abcam, ab6885

### Flow cytometry

3 × 10^5^ HEK293T cells were co-transfected with 2000 ng of *EGFP-LC3B* and 2000 ng of either pCGCG-NL4-3 *nef*, SIV_mac_ 239 *nef*, Chimeras I, II, III, IV, I.I, I.II, I.III, pCI-NL4-3 *nef*-HA, or pairwise amino acid mutants of Nef. 2000 ng of the full-length proviruses of HIV-1 transmitted/founder viruses CH077 (pCH077.t/2627), CH106 (pCH106.c/2633), RHPA (pRHPA.c/2635), THRO (pTHRO.c/2626), TRJO (pTRJO.c/2851, CH058 (pCH058.c/2960), Z331M (pZ331M), Z3576 (pZ3576F), 3618 (pZ3618M), and Z4248 (pZ4248M) were used to assess their overall ability to circumvent autophagy. Forty-eight hours post-transfection, cells were trypsinized (ThermoFisher Scientific, #25200), collected in 1 mL of DPBS and centrifuged at 400×*g* for 5 min. After discarding the supernatant, cells were washed and permeabilized for 10 min at 4 °C using 0.05% saponin (Sigma-Aldrich, #47036) in DPBS. Cells were subsequently washed 2 additional times, resuspended in 2 mL DPBS, and centrifuged at 400×*g* for 5 min. Finally, samples were fixed with 2% paraformaldehyde in DPBS. Cells were analyzed using an Attune instrument (ThermoFisher Scientific, Waltham, MA) and a BD Accuri C6 Plus instrument (BD Biosciences, Franklin Lakes, NJ). The data obtained from 50000 events were then processed using FlowJo software (version 10.5.3). Debris and doublets were excluded through FSC and SSC gating, and the percentage of EGFP-positive single cells was calculated for each sample after setting an appropriately gate using an unstained control.

4 × 10^5^ HEK293T or TZM-bl cells were transfected with 3000 ng of either pCI-NL4-3-Nef or pCI-NL4-3-Nef_48–49_. For SERINC5 analyses, HEK293T cells were additionally co-transfected with 500 ng of pcDNA5-SERINC5-HA. Forty-eight hours post-transfection, cells were trypsinized, collected in 1 mL of DPBS and centrifuged at 400×*g* for 5 min. After discarding the supernatant, cells were incubated in 200 μL of blocking buffer (5% FBS-containing DPBS) for 10 min at room temperature prior to staining using antibodies specific for human APC-HLA-ABC (MHC-I), or FITC-CD4 (Table 1) for 20 min in the dark at room temperature. Cells were then washed twice, resuspended in 2 mL DPBS, and centrifuged at 400×*g* for 5 min. Finally, samples were fixed with 2% paraformaldehyde in DPBS and imaged. For the SERINC5 staining, a primary anti-SERINC5 rabbit antibody that associates with the extracellular domain of SERINC5 was used followed by washes and incubation with an anti-rabbit Alexa-488 secondary antibody (Table 1). Finally, samples were fixed with 2% paraformaldehyde in DPBS. Cells were analyzed using a BD Accuri C6 Plus instrument (BD Biosciences, Franklin Lakes, NJ). The data obtained from 50000 events were then processed using FlowJo software (version 10.5.3). Debris and doublets were excluded through FSC and SSC gating, and the median fluorescence of APC/Alexa488/FITC-positive single cells was calculated for each sample after setting an appropriately gate using an unstained control.

### Immunoprecipitation assays

For the Gag-LC3 immunoprecipitations, 8 × 10^5^ HEK293T cells were transfected with 2000 ng of HIV-1 NL4-3 proviral DNA or p*Gag*-*EGFP* (or the mutants G_2_A-Gag, Ub-Gag and G_2_A/Ub-Gag). For some IPs, 2000 ng of pC3-*EGFP-LC3B* were also included. For the BECN1-BCL2 IPs, 8 × 10^5^ HEK293T cells were transfected with 2000 ng of pcDNA4-BECN1-Flag and 2000 ng of either pCI, pCI-NL4-3-Nef, or pCI-NL4-3-Nef_48–49_. Forty-eight hours post-transfection, cells were washed and lysed using lysis IP buffer supplemented with protease inhibitors and phosphatase inhibitor cocktails 2 and 3. The whole cell lysates were then pre-incubated for 1 h at room temperature with Protein G magnetic beads (New England Biolabs, #S1430S) to pre-clear the samples by removing unspecific binding proteins. Fresh protein G magnetic beads were then pre-coated with the primary antibody of interest for each assay (anti-HIV-1 Gagp55/p24, anti-LC3 clone D11 or anti-BCL2) (Table 1) for 1 h at room temperature. Next, pre-cleared cell lysates were subjected to immunoprecipitation by incubating them with the antibody-coated beads overnight at 4 °C on a rotating platform. Next, beads were washed in lysis IP buffer five times. Finally, washed beads were resuspended in 2× SDS sample buffer and boiled for 5 min prior to their analysis by western blot. The relative binding between our proteins of interest was calculated by densitometric analyses using the software Image Studio (Li-Cor) and Image Lab (BioRad).

### Fluorescence microscopy

2 × 10^4^ HEK293T stably expressing EGFP-LC3 were transfected in sterile tissue culture-treated 8-well slides with 100 ng of HIV NL4-3∆*nef*. Similar assays were performed by providing in trans 100 ng of NL4-3 Nef-HA, 48–49 Nef-HA or HA-GST. Forty-eight hours post-transfection, a set of cells was treated for 4 h with rapamycin at 4 μM. After washing the samples with DPBS (Invitrogen, 14190-144), permeabilization and fixation was achieved by incubating the cells for 10 min in acetone-methanol (1:1) at – 30 °C. Next, cells were incubated for 1 h with the blocking antibody diluent solution (2% fish skin gelatin + 0.1% triton X-100 1× DPBS with 10% goat serum) and incubated 1 more hour with mouse monoclonal anti-Gag p55/p24 primary and rabbit polyclonal anti-HA antibody mix at 1:100 and 1:200 dilution, respectively (Table 1). Subsequently, cells were washed and incubated for another hour with a goat anti-mouse IgG1 secondary antibody conjugated with an Alexa-350 or Alexa-568 fluorophore to stain for Gag using a 1:200 and 1:500 dilution, respectively. HA was visualized with a goat anti-rabbit antibody conjugated with Alexa-568 (Table 1) at a dilution 1:200. For the Gag-LC3 only slides, the nuclei were stained by incubating the samples for 5 min with DAPI (1:5000; Invitrogen, 62248).

For the LC3 puncta studies, 2 × 10^4^ HEK293T cells stably expressing EGFP-LC3 were transfected with 100 ng of HA-GST, NL4-3 Nef-HA, 36–39 Nef-HA or 48–49 Nef-HA. Forty-eight hours later, cells were stimulated with rapamycin (4 μM) for 4 h, prior to microscopy visualization. Cells were stained in an analogous manner as in the assays detailed above, using a mouse monoclonal anti-HA primary antibody at 1:200 dilution and an anti-mouse IgG1 secondary antibody conjugated to Alexa-568 at a 1:500 dilution (Table 1).

Prior to their visualization, the slides were washed using distilled water and mounted using an anti-quenching mounting medium (Vector Laboratories, #3304770). Visualization was performed by confocal microscopy on an Olympus FV3000 microscope and a Lionheart imaging instrument (BioTek, Winooski, VT) using the 60× and 40× objective and the lasers/filter cubes 405, 488, and 561 nm to achieve the excitation of DAPI, GFP and Alexa-568, respectively. After collection, images were processed and analyzed using ImageJ and Photoshop (Adobe), where proportional adjustments of brightness/contrast were applied.

### Statistical analysis

All statistical calculations were performed with a two-tailed unpaired Student T test using Graph Pad Prism version 9.0.0. *P* values ≤ 0.05 were considered statistically significant.

## Supplementary Information

**Additional file 1: Figure S1.** Representative dot plots of the analysis of saponin-resistant EGFP-LC3-II in HEK293T cells transfected with SIV_mac_239 Nef, HIV-1 NL4-3 Nef, or the Nef chimeras I, II, III, or IV. FSC: forward scatter.

## Data Availability

All data generated or analyzed during this study are included in this published article.
