# Supplementary figures and images for: Residues T48 and A49 in HIV-1 NL4-3 Nef are responsible for the counteraction of autophagy initiation, which prevents the ubiquitin-dependent degradation of Gag through autophagosomes
Source: Retrovirology. 2021 Oct 28;18:33. doi: 10.1186/s12977-021-00576-y (PMC8555152; doi:10.1186/s12977-021-00576-y)

FSC

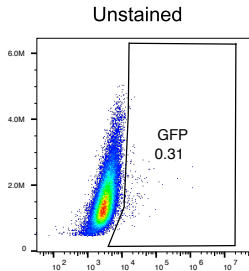

SIV Nef

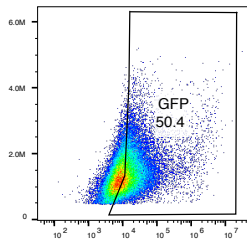

NL4-3 Nef

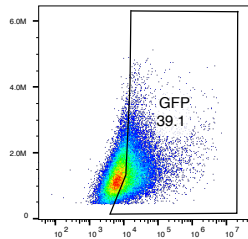

Chimera I

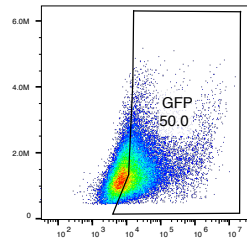

Chimera II

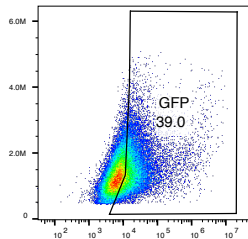

Chimera III

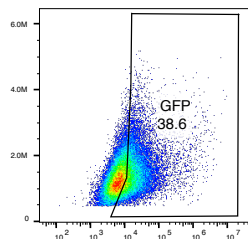

Chimera IV

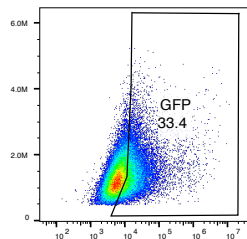

GFP

Supplement: Supplementary file 1 — Additional file 1: Figure S1. Representative dot plots of the analysis of saponin-resistant EGFP-LC3-II in HEK293T cells transfected with SIVmac239 Nef, HIV-1 NL4-3 Nef, or the Nef chimeras I, II, III, or IV. FSC: forward scatter. [file 12977_2021_576_MOESM1_ESM.pdf]
